# Social/economic costs and health-related quality of life in patients with epidermolysis bullosa in Europe

**DOI:** 10.1007/s10198-016-0783-4

**Published:** 2016-04-23

**Authors:** Aris Angelis, Panos Kanavos, Julio López-Bastida, Renata Linertová, Juan Oliva-Moreno, Pedro Serrano-Aguilar, Manuel Posada-de-la-Paz, Domenica Taruscio, Arrigo Schieppati, Georgi Iskrov, Valentin Brodszky, Johann Matthias Graf von der Schulenburg, Karine Chevreul, Ulf Persson, Giovanni Fattore, BURQOL-RD Research Network

**Affiliations:** 1 Department of Social Policy and LSE Health, London School of Economics and Political Science, London, UK; 2 University of Castilla-La Mancha, Talavera de la Reina, Toledo Spain; 3 Red de Investigación en Servicios Sanitarios en Enfermedades Crónicas (REDISSEC), Madrid, Spain; 4 Fundación Canaria de Investigación Sanitaria (FUNCANIS), Las Palmas de Gran Canaria, Spain; 5 University of Castilla-La Mancha, Toledo, Spain; 6 Evaluation and Planning Service at Canary Islands Health Service, Santa Cruz de Tenerife, Spain; 7 Institute of Rare Diseases Research, ISCIII, SpainRDR and CIBERER, Madrid, Spain; 8 National Center for Rare Diseases, Istituto superiore di sanità (ISS), Rome, Italy; 9 Centro di Ricerche Cliniche per Malattie Rare Aldo e Cele Daccò, Istituto di Ricerche Farmacologiche Mario Negri, Ranica (Bergamo), Italy; 10 Institute of Rare Diseases, Plovdiv, Bulgaria; 11 Department of Social Medicine and Public Health, Faculty of Public Health, Medical University of Plovdiv, Plovdiv, Bulgaria; 12 Department of Health Economics, Corvinus University of Budapest, Budapest, Hungary; 13 Centre for Health Economics Research Hannover (CHERH), Leibniz Universität Hannover, Hannover, Germany; 14 URC Eco Ile de France, AP-HP, Paris, France; 15 Université Paris Diderot, Sorbonne Paris Cité, ECEVE, UMRS 1123, Paris, France; 16 INSERM, ECEVE, U1123, Paris, France; 17 The Swedish Institute for Health Economics, Lund, Sweden; 18 Centre for Research on Health and Social Care Management (CERGAS), Bocconi University, Milan, Italy

**Keywords:** Epidermolysis bullosa, Cost-of-illness, Social cost, Health-related quality of life, European Union, Rare disease, I1

## Abstract

**Background:**

The aim of this study was to determine the social/economic costs and health-related quality of life (HRQOL) of patients with epidermolysis bullosa (EB) in eight EU member states.

**Methods:**

We conducted a cross-sectional study of patients with EB from Bulgaria, France, Germany, Hungary, Italy, Spain, Sweden and the United Kingdom. Data on demographic characteristics, health resource utilisation, informal care, labour productivity losses, and HRQOL were collected from the questionnaires completed by patients or their caregivers. HRQOL was measured with the EuroQol 5-domain (EQ-5D) questionnaire.

**Results:**

A total of 204 patients completed the questionnaire. Average annual costs varied from country to country, and ranged from €9509 to €49,233 (reference year 2012). Estimated direct healthcare costs ranged from €419 to €10,688; direct non-healthcare costs ranged from €7449 to €37,451 and labour productivity losses ranged from €0 to €7259. The average annual cost per patient across all countries was estimated at €31,390, out of which €5646 accounted for direct health costs (18.0 %), €23,483 accounted for direct non-healthcare costs (74.8 %), and €2261 accounted for indirect costs (7.2 %). Costs were shown to vary across patients with different disability but also between children and adults. The mean EQ-5D score for adult EB patients was estimated at between 0.49 and 0.71 and the mean EQ-5D visual analogue scale score was estimated at between 62 and 77.

**Conclusion:**

In addition to its negative impact on patient HRQOL, our study indicates the substantial social/economic burden of EB in Europe, attributable mostly to high direct non-healthcare costs.

**Electronic supplementary material:**

The online version of this article (doi:10.1007/s10198-016-0783-4) contains supplementary material, which is available to authorized users.

## Introduction

Epidermolysis bullosa (EB) is a family of rare genetic dermatological conditions. It consists of a group of inherited connective tissue disorders characterised by the absence of a particular cohesion protein in the skin that results in the defective connection of its outer and inner layers (epidermis and dermis) making it fragile [1, 2]. The result is that the top layer of skin does not ‘stick’ securely to the layer beneath it, and, where the two layers separate, a blister develops. EB can be classified into four main types based on the layer of the skin affected: EB simplex (EBS), junctional EB (JEB), dystrophic EB (DEB), and the recently added Kindler syndrome (KS) [1, 3, 4]. Each type can be further subdivided at the molecular level according to the structural gene targeted by the mutation, but also at tissue level, either as generalised (with sites of blistering corresponding to areas where friction is caused by clothing) or localised (where blistering is localised to the hands and feet) [1]. Depending on the severity of the disease, which can vary from benign to life-threatening, symptoms can include skin fragility, blistering of the skin following mild friction or trauma, and blistering of the mucous membranes or internal organs, often leading to a shorter lifespan [3, 5–7]. The prevalence of EB is 5:100,000 live births, with no racial or gender differences [8]. In the EU, the prevalence is estimated to be 2.4:100,000 population [9].

There is currently no cure for EB, and clinical management is focussed on treating the symptoms of the disease [2, 7]. Topical agents and dressings are typically used for the treatment of skin lesions [10], and appropriate follow-up is essential to monitor the patient for a multitude of secondary psychological symptoms, in particular depression, anxiety and behavioural disturbances [3].

The impact of the condition on patient health-related quality of life (HRQOL) has been explored both from a qualitative and a quantitative perspective [2, 3, 5–7, 11–15]. Qualitative results have revealed a high prevalence of psychosocial problems and psychiatric symptoms [3, 13], indicating the importance of providing the appropriate psychological and peer support [2], in tandem with pain management and nursing support, as part of a multidisciplinary approach [14]. More quantitative approaches have assessed different HRQOL dimensions of EB patients using a range of instruments including the dermatology life quality index (DLQI) and the children’s dermatology life quality index (CDLQI) [5], the QOLEB questionnaire [6, 15], the short form-36 (SF-36), Skindex-29, General Health Questionnaire-12 (GHQ-12) and EuroQOL 5 dimensions (EQ-5D) questionnaires [7], while others have also looked at the burden to carers [7] and parents [12]. Overall, they have shown impaired HRQOL for both the patients [5, 7, 11] and their carers [7, 12]. However, there is an absence of economic and cost information related to the disease and its impact. As part of the BURLQOL-RD initiative [16], this study used patient-level primary data collected across eight EU Member States (Bulgaria, France, Germany, Hungary, Italy, Spain, Sweden and the UK) to estimate the social and economic cost burden of EB in terms of direct healthcare, direct non-healthcare, and loss of productivity (indirect) costs, and also report the loss in HRQOL, for both patients and their caregivers.

## Methodology

### Research design and sample

This was a bottom-up, cross-sectional, study of non-institutionalised patients diagnosed with EB who received outpatient care. Because of the lack of patient registries at national level, subjects were recruited with the assistance of the EB associations across the study countries based on the associations’ membership. The survey was anonymous and the patients were contacted by their patient organisation. An EB diagnosis, non-institutionalised status and membership with the respective EB national patient association were the criteria for patient eligibility. Questionnaire responses received by the research team had no identification information (i.e. name, identification, address/postcode, e-mail or telephone). All patients and caregivers were informed about the study objective, data confidentiality and were asked to indicate their understanding of the study conditions and agreement to participate. The study protocol was submitted to the London School of Economics (LSE) Research Ethics Committee and received an exemption.

Following identification of the patient sample, patient associations administered questionnaires electronically or by post to eligible patients between September 2011 and April 2013; however, the recruitment period did not exceed 6 months in any of the countries. The questionnaires were distributed by e-mail and post through patient organisations. Demographic, clinical and resource use data were collected from EB patients and their caregivers.

### Costing methodology

We used the prevalence approach to estimate the resources used and, subsequently, costs incurred from a societal perspective. Disease prevalence takes into account all direct healthcare resources used for prevention, treatment and rehabilitation, other non-healthcare resources used (formal and informal care), and lost labour productivity within a given year (in a population or in a sample of patients) as a consequence of the illness considered. Prevalence-based cost-of-illness analysis has the advantage of incorporating measurements of total annual healthcare expenditure, which is particularly relevant for chronic conditions requiring long-term treatment such as EB. In this context, a bottom-up costing approach was used to estimate total and average annual costs [17].

Data on resource utilisation were collected for each patient and, where appropriate, for the caregiver as well. To estimate resource utilisation, the questionnaire solicited information regarding the 6-month period prior to the study (12 months for hospital admissions), and extrapolated the data to the entire year. We considered 6 months to be an appropriate recall period. Productivity losses were calculated using data collected on reductions in patient and caregiver working time (temporary and permanent sick leave or early retirement). Non-professional caregivers were also asked about informal care time.

### Direct healthcare costs

Direct medical costs were derived from healthcare utilisation. The cost of resources used by patients was calculated based on the relevant unit costs and the average utilisation per patient in the sample. Information about the number of hospital admissions, the number of emergency visits and data for the volume of outpatient care (rehabilitation, medical tests and examinations, visits to health professionals and home medical care) were generated from the questionnaires.

Unit costs were obtained from different databases used in Europe on healthcare costs, and any remaining data gaps were filled in using additional publicly available resources (see Annex II of the ESM). To derive the annual cost per patient, unit costs were multiplied by the respective resource quantities, using 2012 as the reference year. Similarly, resource utilisation information relating to use of prescription drugs and medical support devices was obtained from the questionnaires. When no information concerning the number of units per pack was available, we assumed the largest dispensing pack for prescription drugs. Prescription drug unit costs were also obtained from government databases (see Annex II of the ESM), whereas unit costs for medical support devices were obtained from major electronic commerce websites.

### Direct non-healthcare costs

Direct non-medical costs were quantified by aggregating three items: non-healthcare transportation, social care services (formal care), and caregiver’s time (informal care provided by non-professional caregivers, who are often relatives, but could also be friends or neighbours). Informal care concerned the time spent helping the patient with their basic activities of daily living (ADL), and the time spent helping with necessary instrumental activities of daily living (IADL) (recall method). As a conservative criterion, and for preventing conjoint production, we have censored the time of care to a maximum of 16 h per day (114 h per week) when the time of care reported exceeded this figure. To cost care hours, the proxy good method was used, whereby time was valued as an output, and the care provided by the informal caregiver was valued such that if they did not provide these services, they would have instead been provided by a professional caregiver [18, 19]. Data on formal (paid) care provided by professional caregivers and other social services were obtained from the questionnaires and reported in the relevant category.

### Productivity losses

Productivity losses were accounted for by converting physical units (days of sick leave and early retirement) into monetary units using the human capital approach [20]. Worker gross average earnings were used to proxy productivity losses (see Annex II of the ESM). Therefore, our calculations were based on average gross wage figures in the Wage Structure Surveys by the National Statistics Institutes of the participating countries. Annual labour productivity losses were estimated for the year 2012.

### Patient and caregiver outcomes

Patient and caregiver outcomes were obtained via the EQ-5D [21], the Barthel index [22] and the Zarit burden interview [23]. The EQ-5D is a generic instrument of HRQOL commonly used in economic evaluations and routinely included in health technology assessments (HTAs). It has five dimensions (mobility, self-care, everyday activities, pain/discomfort and anxiety/depression). The values and utilities are assigned a score on a scale where 0 corresponds to death and 1 corresponds to perfect health, with negative values being possible.

The second part of the EQ-5D consists of a vertical 20-cm, 0–100 visual analogue scale (VAS), where 0 represents the worst and 100 represents the best imaginable health state. Respondents mark a point on the scale to reflect their overall perception of health on the day of the interview [21]. Evaluations of these health states have been reported for the general population [24].

The Barthel index is widely used to assess physical disability. It measures the ability of a person to perform ten basic activities of daily living (ADL), and produces a quantitative estimate of the subject’s degree of dependence [22]. Total possible scores range either from 0 to 20 or from 0 to 100, with lower scores indicating increased disability. All scores were converted into the 0–100 range. A score of 91–99 shows mild dependence, 61–90 moderate dependence, 21–60 severe dependence and < 21 complete dependence [25]. Following receipt of completed questionnaires, patients were grouped into two categories: lower (i.e. no or mild) disability, defined as having a Barthel index score between 91 and 100, and higher (i.e. moderate, severe or complete) disability, for Barthel Index scores lower than 91.

Lastly, the Zarit Burden Interview (22-item version) measures the subjective burden among caregivers. Each item is a statement that the caregiver is asked to respond to using a five-point scale, with options ranging from 0 (never) to 4 (nearly always). The total score ranges from 0 to 88, where scores under 21 correspond to little or no burden, and scores over 61 represent severe burden [23].

## Results

A total of 339 questionnaires were collected in the eight countries from people with EB, 135 of which were excluded because the information they contained was insufficient or inadequate. Therefore, the valid sample totalled 204 [8 Bulgaria, 37 France, 15 Germany, 6 Hungary, 35 Italy, 54 Spain, 6 Sweden and 43 United Kingdom (UK)].

The main characteristics of the sample are summarised in Table 1. Of the total patient sample (*n* = 204), more than half of participants were adults (*n* = 121), and their average age was 26.7 years, with considerable uniformity in the sample across countries in terms of average age, the only outliers being Bulgaria, where patients were younger (average 14.6 years) and the UK, where the patients were older (average 34.3 years). Mean age for children in the sample (*n* = 93) was 7.2 years. There was a higher prevalence of female patients in the sample, accounting on average for 59.8 % of respondents, with Hungary and Sweden being outliers with an average of 33.3 and 83.3 % males, respectively. Most patients, 60.3 % (*n* = 123), did not require a caregiver.

**Table 1:** Demographic characteristics of the study participants (patients = 204; caregivers = 81)

	Bulgaria	France	Germany	Hungary	Italy	Spain	Sweden	United Kingdom	Average
**Patients**									
No.	8	37	15	6	35	54	6	43	
Sex, males (%)	37.5	48.6	26.7	66.7	48.6	38.9	16.7	32.6	40.2
Average age years (SD)	14.6 (19.0)	27.8 (21.0)	28.6 (19.1)	21.3 (18.9)	20.6 (15.6)	25.5 (19.8)	27.0 (18.3)	34.3 (19.9)	26.7 (19.6)
Disease subtype (%)^a^									
Simple generalised	0.0	40.5	26.7	0.0	14.3	18.5	16.7	27.9	23.0
Simple localised	0.0	8.1	26.7	0.0	2.9	9.3	50.0	34.9	15.2
Junctional generalised	37.5	8.1	6.7	0.0	8.6	3.7	0.0	2.3	6.4
Junctional localised	0.0	0.0	13.3	0.0	0.0	0.0	0.0	0.0	1.0
Dystrophic generalised	12.5	24.3	26.7	66.7	65.7	48.1	0.0	20.9	37.3
Dystrophic localised	25.0	18.9	0.0	16.7	8.6	11.1	0.0	14.0	12.3
**Caregivers (main)**									
No.	5	9	6	2	23	26	1	9	
Sex, males (%)	0.0	0.0	16.7	0.0	21.7	15.4	n/a	11.1	13.6
Average age, years (SD)	35.4 (8.5)	41.6 (8.7)	40.0 (10.8)	39.0 (5.7)	36.8 (18.4)	47.7 (15.3)	n/a	41.5 (14.0)	41.7 (15.2)
Relationship to patient (%)^b^									
Parent	n/a	55.6	66.7	50.0	21.7	92.3	n/a	44.4	53.1
Other relative	n/a	11.1	0.0	0.0	65.2	7.7	n/a	11.1	23.5
Partner or other	n/a	11.1	33.3	50.0	8.7	3.8	n/a	33.3	12.3
Employment status (%)^c^									
Employed	n/a	44.4	50.0	50.0	47.8	42.3	n/a	33.3	40.7
Retired	n/a	0.0	0.0	0.0	13.0	23.1	n/a	0.0	11.1
Houseworker	n/a	22.2	16.7	50.0	21.7	34.6	n/a	55.6	28.4
Other	n/a	11.1	33.3	0.0	13.0	3.8	n/a	0.0	8.6
Average dedication, hours per week (SD)^d^	89.8 (29.5)	34.3 (36.7)	80.3 (37.0)	78.0 (48.1)	59.4 (36.1)	80.8 (30.7)	0.0 (n/a)	75.1 (50.2)	68.4 (38.8)

*SD* standard deviation

^a^Disease subtype percentages for Bulgaria, Hungary, Spain, Sweden (and as a result all countries average) do not add up to 100 % because of missing data points

^b^Relationship to patient percentages for France, Italy, UK (and as a result all countries average) do not add up to 100 % because of missing data points. Percentages for Bulgaria and Sweden are unknown because of missing coding

^c^Employment status percentages for France, Italy, UK (and as a result all countries average) do not add up to 100 % because of missing data points. Percentages for Bulgaria and Sweden are unknown because of missing coding

^d^Sweden’s standard deviation (SD) for dedication, hours per week, is not available because there was only a single carer (i.e. *n* = 1)

The average age of caregivers, for those patients that had one, was 40.3 years, and female caregivers dominated the sample (86.4 %). Taking into account some missing data, about half of the caregivers across the entire sample were parents to the patients (53.1 %), followed by other family relatives (23.5 %) and partners or others (12.3 %). Across all countries, a substantial proportion of caregivers was in paid employment other than caregiving (40.7 %), with a considerable proportion being involved with domestic activities (28.4 %) and a smaller proportion being retired (11.1 %). The total average time spent caregiving by the main caregiver (if there was at least one caregiver) was estimated at between 34 h per week (France) and 90 h per week (Bulgaria).

With regards to HRQOL, the mean EQ-5D index scores (TTO tariff) ranged from 0.49 (Hungary) to 0.71 (Sweden) (Table 2). The EQ-5D VAS scores ranged from 62 (Spain) to 77 (Bulgaria) (Table 2). These scores are noticeably lower than scores reported in the general population across the study countries [24].

**Table 2:** Quality of life characteristics of the study participants

	Bulgaria	France	Germany	Hungary	Italy	Spain	Sweden	UK	Average
**EQ-5D index score**									
Adult patients that completed EQ 5D (*n* = 111), mean (SD)	0.608 (0.057)	0.607 (0.213)	0.595 (0.293)	0.492 (0.317)	0.504 (0.279)	0.606 (0.282)	0.707 (0.087)	0.563 (0.340)	0.579 (0.280)
General UK population (adult patients’ age-adjusted), mean (SD)	0.85 (0.25)	0.91 (0.16)	0.91 (0.16)	0.91 (0.16)	0.93 (0.15)	0.91 (0.16)	0.91 (0.16)	0.91 (0.16)	0.91 (0.16)
Main caregivers that completed EQ 5D (*n* = 68), mean (SD)^a^	0.127 (0.535)	0.734 (0.184)	0.652 (0.412)	0.758 (0.015)	0.786 (0.300)	0.738 (0.284)	n/a	0.675 (0.170)	0.696 (0.334)
General UK population (caregivers’ age-adjusted), mean (SD)	0.91 (0.16)	0.91 (0.16)	0.91 (0.16)	0.91 (0.16)	0.91 (0.16)	0.85 (0.25)	n/a	0.91 (0.16)	0.91 (0.16)
**VAS**									
Adult patients that completed VAS (*n* = 111), mean (SD)	77.5 (20.6)	68.7 (18.3)	63.2 (21.8)	71.7 (10.4)	63.1 (15.1)	62.4 (23.1)	77.5 (20.6)	67.9 (23.9)	66.2 (20.7)
General UK population (adult patients’ age-adjusted), mean (SD)	82.0 (18.2)	86.6 (13.8)	86.6 (13.8)	86.6 (13.8)	86.6 (13.8)	86.6 (13.8)	86.6 (13.8)	86.6 (13.8)	86.6 (13.8)
Main caregivers that completed VAS (*n* = 66), mean (SD)^b^	64.8 (17.6)	67.0 (21.6)	66.3 (13.8)	70.0 (28.3)	76.8 (14.5)	75.1 (19.7)	n/a	69.2 (20.4)	73.1 (18.0)
General UK population (caregivers’ age-adjusted), mean (SD)	86.6 (13.8)	86.6 (13.8)	86.6 (13.8)	86.6 (13.8)	86.6 (13.8)	82.0 (18.2)	n/a	86.6 (13.8)	86.6 (13.8)
Adolescent patients that completed VAS (*n* = 41), mean (SD)	70.0 (14.1)	59.3 (23.9)	73.8 (22.9)	70.0 (28.3)	51.7 (14.6)	69.0 (19.1)	77.5 (24.7)	73.0 (22.8)	65.0 (20.4)
**Zarit scale** **(** ***n*** ** = 68), mean (SD)** ^c^	n/a	35.3 (20.8)	38.3 (8.9)	n/a	31.6 (13.9)	30.3 (11.7	n/a	18.5 (10.5)	30.5 (13.7)
**Barthel index** **(** ***n*** **= 149), mean (SD)** ^d^	56.3 (13.1)	96.9 (9.0)	85.8 (21.0)	84.0 (16.4)	73.3 (26.0)	81.4 (22.7)	90.8 (20.1)	91.9 (14.7)	85.2 (21.0)

*SD* standard deviation; * VAS* visual analog scale

^a^EQ-5D index score for Sweden’s main caregivers is not available because of missing data point

^b^VAS score for Sweden’s main caregivers is not available because of missing data point

^c^Zarit scale scores for Bulgaria, Hungary and Sweden are not available because of missing data points

^d^Barthel scores for Sweden and UK were re-escalated from a 20-point scale to a 100-point scale

Over three-quarters of caregivers (*n* = 68) completed the HRQOL portions of the questionnaire. Mean EQ-5D index scores for caregivers ranged from 0.127 (Bulgaria) to 0.79 (Italy) (Table 2). Mean EQ-5D VAS scores for caregivers (*n* = 66) ranged from 64.8 (Bulgaria) to 76.8 (Italy) (Table 2).

The average Barthel index (BI) score of patients from France and the United Kingdom reflected mild dependence (96.9 and 91.9, respectively) (Table 2). The average BI score of patients from Germany, Hungary, Italy, and Spain reflected moderate dependence (85.8, 84.0, 73.3 and 81.4, respectively). The average BI score of patients from Sweden lay on the boundary between mild and moderate dependence (90.8). The average BI score of patients from Bulgaria reflected severe dependence (56.3). Lastly, the average BI score across all countries reflected moderate dependence (85.2).

The burden for caregivers was mild in the UK (average Zarit scale score of 18.5) and moderate in France, Germany, Italy and Spain (average Zarit scale scores of 35.3, 38.3, 31.6, 30.3, respectively) (Table 2). Across all countries, on average, the burden for caregivers was moderate (Zarit scale score of 30.5). Average annual cost per patient in 2012 was estimated at €17,671, €14,931, €46,116, €9809, €49,233, €43,137, €9509, and €19,758, for patients in Bulgaria, France, Germany, Hungary, Italy, Spain, Sweden and the UK, respectively (Table 3). The largest cost components were non-healthcare costs in all the countries. Formal care had a minor weight in all countries analysed except in Sweden. Meanwhile, informal care had a major weight in Spain, the UK, France, Hungary, Bulgaria, Germany and Italy, with the exception being Sweden, where informal care was virtually non-existent in this sample of EB patients (Table 3). Loss of labour productivity is a minor item in all countries with the exception of Germany and the UK.

**Table 3:** Average annual costs per patient (SD), all patients (2012, €)

	Bulgaria	France	Germany	Hungary	Italy	Spain	Sweden	UK	Average
Drugs	46 (78)	263 (1310)	36 (50)	48 (57)	2794 (1664)	235 (457)	3 (8)	50 (66)	606 (1353)
Medical tests	50 (66)	104 (182)	187 (332)	25 (34)	95 (247)	190 (316)	9 (23)	197 (461)	144 (312)
Medical visits	271 (295)	868 (1490)	4378 (6542)	74 (91)	1825 (1844)	1587 (3837)	1168 (2676)	1945 (3199)	1670 (3301)
Hospitalizations	990 (2282)	2238 (7335)	3547 (5463)	218 (478)	5420 (15,334)	2042 (6197)	0 (0)	401 (2028)	2267 (8060)
Health material	2232 (457)	1371 (1701)	777 (1880)	44 (64)	549 (1232)	390 (1371)	298 (691)	1532 (1137)	924 (1451)
Healthcare transport	0 (0)	10 (41)	153 (340)	10 (25)	4 (22)	66 (423)	0 (0)	20 (93)	36 (242)
**Direct healthcare costs**	3589 (2604)	4853 (9806)	9076 (11,664)	419 (490)	10,688 (15,956)	4511 (8584)	1478 (3387)	4146 (4786)	5646 (10,062)
Social health service	256 (590)	2975 (10020)	2243 (7322)	744 (1822)	1896 (5197)	598 (1842)	2290 (5609)	15 (98)	1290 (5383)
Professional carer	0 (0)	124 (556)	0 (0)	0 (0)	403 (1661)	0 (0)	5088 (12,463)	716 (4694)	392 (3107)
Non-healthcare transport	60 (149)	144 (362)	156 (319)	109 (211)	135 (415)	58 (114)	71 (174)	31 (76)	90 (262)
Main informal carer	8630 (7925)	4346 (11,913)	21,779 (31,422)	6529 (11,466)	24,227 (31,300)	27,634 (32,470)	0 (0)	10,107 (24,367)	16,522 (27,481)
Other informal carers	4870 (6865)	1294 (5122)	5604 (12,715)	2009 (4921)	10,790 (18,888)	8534 (18,299)	0 (0)	860 (4087)	5188 (13,598)
**Direct non-healthcare costs**	13,817 (13,544)	8884 (22,864)	29,781 (44,834)	9390 (16,554)	37,451 (45,216)	36,824 (44,974)	7449 (18,246)	11,729 (26,655)	23,483 (37,938)
Productivity loss	265 (750)	1194 (4076)	53 (144)	0 (0)	428 (2189)	115 (718)	582 (1426)	83 (303)	369 (2036)
Early retirement	0 (0)	0 (0)	7206 (14,919)	0 (0)	666 (2208)	1688 (6073)	0 (0)	3800 (10,600)	1892 (7292)
**Indirect total**	265 (750)	1194 (4076)	7259 (14,892)	0 (0)	1094 (3013)	1803 (6083)	582 (1426)	3884 (10,574)	2261 (7478)
**TOTAL**	17,671 (14,923)	14,931 (29,947)	46,116 (55,976)	9809 (16,315)	49,233 (48,623)	43,137 (49,864)	9509 (21,397)	19,758 (33,496)	31,390 (43,577)

*SD* standard deviation

**Fig. 1:**
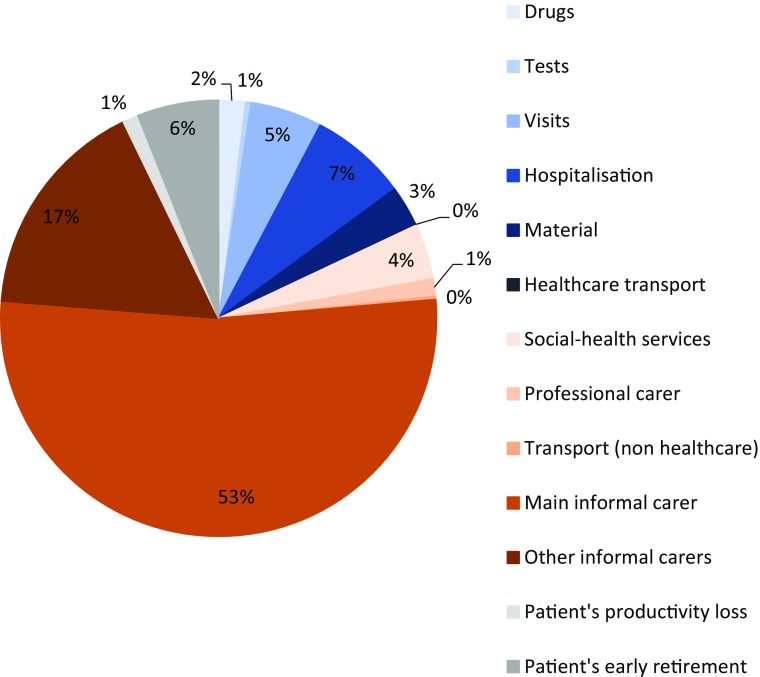
Mean costs per average epidermolysis bullosa (EB) patient broken down by type of cost (2012, €)

**Fig. 2:**
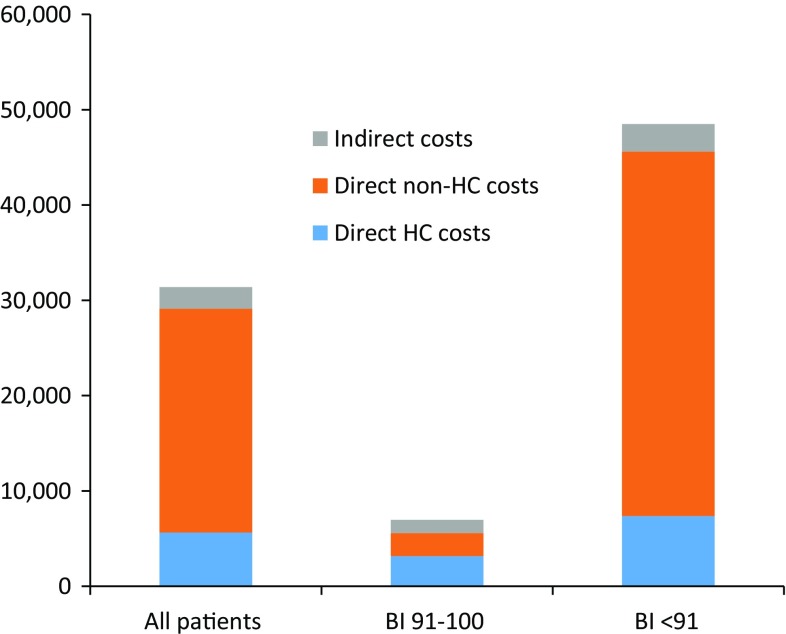
Direct healthcare (HC), direct non-healthcare (non-HC) and indirect costs according to patient disability (2012, €)

The average annual cost per patient across all countries was estimated at a total of €31,390, out of which €5646 accounted for direct health costs (18.0 % of total), €23,483 accounted for direct non-healthcare costs (74.8 % of total), and €2261 accounted for indirect costs (7.2 % of total) (Fig. 1). Patients with no or mild disability (BI score 91–100) had a lower average annual cost of €6961 (direct healthcare costs of €3170, direct non-healthcare costs of €2396, indirect costs of €1394) whereas patients with moderate or severe disability (BI score < 91) had a higher average annual cost of €48,491 (direct healthcare costs of €7378, direct non-healthcare costs of €38,244, indirect costs of €2868) (Fig. 2).

When looking specifically at the costs incurred by adults (121 patients), data were included in the analysis based on the sample sizes of all countries. Mean annual costs ranged from €1003 per patient in Sweden to €35,857 in Italy (Table 4). Direct healthcare costs ranged from €129 per patient in Sweden to €11,025 in Italy. Direct non-healthcare costs ranged from €43 per patient in Bulgaria to €22,816 in Italy. Loss of labour productivity costs ranged from €0 per patient in Hungary to €12,098 in Germany (Table 4).

**Table 4:** Average annual costs per patient, adult patients (2012, €)

	Spain (*n* = 29)	UK (*n* = 33)	France (*n* = 22)	Bulgaria (*n* = 2)	Hungary (*n* = 3)	Germany (*n* = 9)	Sweden (*n* = 4)	Italy (*n* = 19)
Drugs	239	40	53	93	73	25	5	3015
Medical tests	190	197	104	50	25	186	9	95
Medical visits	626	1689	437	465	32	4467	96	2289
Hospitalizations	1494	474	2037	63	396	2956	0	4642
Health material	559	1339	1126	2147	42	25	20	984
Healthcare transport	11	13	8	0	20	144	0	0
**Direct healthcare costs**	**3117**	**3752**	**3765**	**2817**	**589**	**7804**	**129**	**11,025**
Professional carer	0	933	71	0	0	0	0	743
Non-healthcare transport	45	19	75	5	196	88	0	211
Social services	897	19	2322	38	0	602	0	3478
Main informal carer	11,278	2442	778	0	9375	6069	0	12,052
Other informal carers	5282	0	403	0	4018	4745	0	6333
**Direct non-healthcare costs**	**17,502**	**3413**	**3649**	**43**	**13,589**	**11,504**	**0**	**22,816**
Productivity loss	214	109	2008	1061	0	88	873	788
Early retirement	3143	4952	0	0	0	12,011	0	1227
**Indirect costs**	**3357**	**5060**	**2008**	**1061**	**0**	**12,098**	**873**	**2016**
**TOTAL COSTS**	**23,976**	**12,226**	**9421**	**3921**	**14,178**	**31,406**	**1003**	**35,857**

For the paediatric patients, 83 were included in the analysis based on the sample sizes of all countries. Mean annual costs ranged from €5441 per patient in Hungary to €68,181 in Germany (Table 5). Direct healthcare costs ranged from €249 per patient in Hungary to €10,985 in Germany. Direct non-healthcare costs ranged from €5192 per patient in Hungary to €59,236 in Spain (Table 5).

**Table 5:** Average annual costs per patient, paediatric patients (2012, €)

	Spain (*n* = 25)	UK (*n* = 10)	France (*n* = 15)	Bulgaria (*n* = 6)	Hungary (*n* = 3)	Germany (*n* = 6)	Sweden (*n* = 2)	Italy (*n* = 16)
Drugs	232	84	571	31	22	51	0	2532
Medical tests	190	197	104	50	25	186	9	95
Medical visits	2703	2790	1498	206	115	4243	3313	1275
Hospitalizations	2678	161	2532	1298	40	4433	0	6344
Health material	195	2169	1731	2261	47	1904	854	32
Healthcare transport	130	43	12	0	0	167	0	8
**Direct healthcare costs**	**6128**	**5443**	**6448**	**3846**	**249**	**10,985**	**4176**	**10,286**
Professional carer	0	0	203	0	0	0	15,264	0
Non-healthcare transport	73	71	246	79	22	257	213	45
Social services	251	0	3.933	329	1488	4704	6870	18
Main informal carer	46,606	35,400	9580	11,506	3683	45,343	0	38,685
Other informal carers	12,306	3698	2601	6494	0	6892	0	16,083
**Direct non-healthcare costs**	**59,236**	**39,169**	**16,563**	**18,408**	**5192**	**57,196**	**22,346**	**54,831**
Productivity loss	0	0	0	0	0	0	0	0
Early retirement	0	0	0	0	0	0	0	0
**Indirect costs**	**0**	**0**	**0**	**0**	**0**	**0**	**0**	**0**
**TOTAL COSTS**	**65,364**	**44,612**	**23,012**	**22,254**	**5441**	**68,181**	**26,522**	**65,117**

## Discussion

Comparing HRQOL and economic burden across rare diseases helps to inform priority setting for healthcare resource allocation [4, 11, 15, 26]. Our study purports to be the first of its kind to provide comprehensive information on the costs of EB across several countries, and adds to the existing literature on EB cost and HRQOL impact.

The average EQ-5D index score for adult patients with EB was estimated at between 0.49 (Hungary) and 0.71 (Sweden). Patient HRQOL was not similar across the countries, but consistently lower than general population reference values.

Our results suggest that individuals living with EB have markedly lower HRQOL than the general population and, by extension, their caregivers also suffer decreased HRQOL.

This analysis highlights the importance of studying the economic consequences of EB and interpreting the results in an international context. The results of our analysis provide insights into the distribution of the costs of EB and the impact of EB on national expenditures for healthcare. We show that, in 2012, the estimated average annual cost was €17,671, €14,931, €46,116, €9809, €49,233, €43,137, €9509, and €19,758, for patients in Bulgaria, France, Germany, Hungary, Italy, Spain, Sweden and the UK, respectively. The average annual cost across all countries was estimated at €31,390 per patient, with the highest proportion of costs being attributed to direct non-healthcare (74.8 % of total), followed by direct healthcare (18.0 % of total) and productivity loss (7.2 %). Patients with a lower disease severity (as categorised through their BI scores, 91–100) were associated with a lower average cost of €6961 per patient, compared to patients with a higher disease severity (BI score < 91) that were associated with a higher average cost of €48,491.

The high costs of informal care underpin the social economic burden of EB, accounting for more than half of total costs. The clear divide and hidden social costs of EB are relevant to policy-makers, especially when contemplating the impact across tiers of family income [7].

We found that EB had a consistent impact on the HRQOL of patients and their caregivers regardless of the country. In adults, direct healthcare costs, especially hospitalisations, medical visits and health material, represented the vast majority of costs, while in children, medical visits, hospitalisations and direct non-healthcare informal costs, i.e. caregivers’ dedication time, were especially predominant.

A recently published study analysing the socioeconomic impact of ten rare diseases identified no available costing studies of EB [27], apart from an old study that examined the cost-effectiveness of the “Kozak protocol” in treating nine children at a hospital in the Canadian province of Ontario; however, due to a response indicating some serious data discrepancies, these costs were not studied in depth [28, 29].

The only other quantitative study to quantify the HRQOL burden of EB in the UK used the Dermatology Life Quality Index (DLQI) in a Scottish population [5]. HRQOL loss due to EBS and DEB was observed to be similar to severe psoriasis and atopic eczema [5], where children ranked worse than adults. The DLQI was criticised for underestimating the impact of more severe types of EB by proposing questions on activities that may naturally be incompatible with their daily lives (i.e. sports, gardening, shopping) [4–6, 11, 26]. There are other qualitative studies, however, that have investigated particular aspects of the disease, such as the HRQOL impact of living with a gastronomy tube [30, 31], living with wounds [14], dressings and bandages [32] and their associated psychosocial impact [2]. Elsewhere, worse median EQ-5D index scores were reported for children than adults, and the occurrence of problems with mobility, self-care, usual activities and pain/discomfort was more common in children, with the exception of anxiety/depression [7]. No significant differences were observed in mean VAS scores between children and adults, but the mean adult score of 62 (±23) is comparable to our adult and adolescent scores of 66.2 (±20.7) and 65.0 (±20.4). By assessing changes in usual activities, the EQ-5D questionnaire could also succumb to the same flaws as the DLQI; consequently HRQOL values presented in our study could be underestimates. More concerned with levels of dependence in performing ADLs, the Barthel index is less likely to be hindered by the aforementioned methodological issue. Fine et al. [11] surveyed ADL scores in a sample of children, albeit on a different scale than the Barthel index, and observed that most had partial dependence on a caregiver.

Administering both the SF-36 and the Skindex-29 (dermatology specific) questionnaires has been suggested as the best option to evaluate HRQOL in EB patients, striking an adequate balance between disease specificity, conceptual validity, and comparability across diseases [4, 33]. Following this practice, one study noted that women with EB had worse HRQOL compared to men [7], and a second study classified individuals with JEB, RDEB and DDEB as having some of the poorest HRQOL amongst dermatological diseases; the milder EBS ranked close to atopic dermatitis [15]. An EB specific quality of life questionnaire (EBQOL) was developed and validated across populations, although it does fail to fully quantify psychological burden [6, 15].

Qualitative studies have attempted to describe the determinants of the lower HRQOL among patients with EB and the wider social impact this disease may have on patients and families alike, including the likely co-morbidities that may emerge as a result. For children, the main impediments were itchy skin, pain, limitations to participation in childhood activities (i.e. sports, outdoor activities), and the resulting social isolation [4, 5, 12]. Parental and family support was crucial [3, 12], but caring for a child was observed to adversely affect the parents’ marriage and decrease their likelihood of having another child [34]. The burden imposed on caregivers was related to the severity of the condition [7, 34], and was affected by perceptions of the child’s pain, the stigma associated with being different, and limitations to family activities [12]. These factors are likely to have contributed to the lower HRQOL scores reported by the caregivers in our sample.

As in children, the daily limitations on adult patients due to pain and blisters were important [12], and the social impact of EB was of significant concern [13]. Patients were said to experience distress because of the life-long, hereditary nature of the disease, as well as the social isolation, discrimination and anxiety related to the visibility of EB [2, 3, 13, 15]. In a study aiming to conduct a psychosocial and psychiatric evaluation, 82 % of patients had a deteriorated quality of life using the DLQI, and 80 % of patients experienced psychiatric symptoms for which severity was not related to EB [3].

Our study is not without limitations. We did not consider EB type classification when calculating HRQOL and cost burden. As such, even though disability status does capture some of the variation in disease severity, conclusions drawn from our subgroup analysis are restricted to broad comparisons unless the Barthel index scores of EB types are known. In addition, attributing a general HRQOL detriment to EB is complex due to the heterogeneity of severity across and within categories [1, 2, 6]. The Birmingham Epidermolysis Bullosa Severity (BEBS) score could have allowed for stratification of patients based on disease severity to provide more detailed cost estimates [10]. Though there are EB (EBQoL) and dermatology-specific (Skindex-29) HRQOL questionnaires, we used the EQ-5D as it has been deemed a valid cross-sectional, generic measure of health outcomes in rare diseases and is commonly used as the basis for economic evaluations [7, 35]. However, it does appear pertinent to argue for more accurate disease-specific tools and their wider use in the future in terms of capturing some of the salient features of individual diseases and the impact they may exert on patients, which standard tools, such as the EQ-5D, may not be able to capture [36]. A small sample size and recruitment of solely patient volunteers could have introduced a selection bias to the study, causing a preference for patients with less severe illness, as they were more likely not to be hospitalised and to be in contact with the association. If this were true, we would have underestimated the economic burden, as the high treatment costs related to hospitalisation and long-term care could not have been considered. Furthermore, recall biases are non-negligible when conducting questionnaire-based studies and, like other HRQOL studies on EB, cross-sectional data was used; restrictions as to the scope and means of our study made the collection of longitudinal data prohibitive, although this could have captured patient adaptation to their diseased state [4].

The limitations of cost-of-illness studies outlined above are generally trumped by their utility alongside population-level data in helping to formulate health policies. By adopting a bottom-up and annual approach for this study, these first estimates of the costs of EB across eight EU member states are likely to be complete and realistic.

## Conclusion

The social/economic burden of EB, shared between the high direct non-healthcare costs resulting from informal care use and the loss of labour productivity, reinforce the importance of not restricting cost analysis to direct healthcare costs. Future public policy decisions and interventions for EB or other rare diseases, at a national and EU level, should aim to take patient level cost disparities and HRQOL effects into account.

## Electronic supplementary material

Below is the link to the electronic supplementary material.

Supplementary material 1 (DOCX 16 kb)

Supplementary material 2 (DOC 41 kb)

## References

[1] Fine J-D (2008). The classification of inherited epidermolysis bullosa (EB): report of the third international consensus meeting on diagnosis and classification of EB. J. Am. Acad. Dermatol..

[2] Williams EF, Gannon K, Soon K (2011). The experiences of young people with epidermolysis bullosa simplex: a qualitative study. J Health Psychol..

[3] Margari F (2010). Psychiatric symptoms and quality of life in patients affected by epidermolysis bullosa. J. Clin. Psychol. Med. Settings.

[4] Pagliarello C, Tabolli S (2010). Factors affecting quality of life in epidermolysis bullosa. Expert Rev. Pharmacoecon. Outcomes Res..

[5] Horn HM, Tidman MJ (2002). Quality of life in epidermolysis bullosa. Clin. Exp. Dermatol..

[6] Frew JW (2009). Quality of life evaluation in epidermolysis bullosa (EB) through the development of the QOLEB questionnaire: an EB-specific quality of life instrument. Br. J. Dermatol..

[7] Tabolli S (2009). Quality of life in patients with epidermolysis bullosa. Br. J. Dermatol..

[8] NIH. Genetic home reference: epidermolysis bullosa simplex (2007) [cited 2011 May 6th]. http://ghr.nlm.nih.gov/condition/epidermolysis-bullosa-simplex

[9] Orphanet Report Series. Prevalence of rare diseases: bibliographic data. 2013

[10] Moss C, Wong A, Davies P (2009). The Birmingham epidermolysis bullosa severity score: development and validation. Br. J. Dermatol..

[11] Fine JD (2004). Assessment of mobility, activities and pain in different subtypes of epidermolysis bullosa. Clin. Exp. Dermatol..

[12] van Scheppingen C (2008). The main problems of parents of a child with epidermolysis bullosa. Qual. Health Res..

[13] Dures E (2011). The psychosocial impact of epidermolysis bullosa. Qual. Health Res..

[14] Adni T, Martin K, Mudge E (2012). The psychosocial impact of chronic wounds on patients with severe epidermolysis bullosa. J. Wound Care..

[15] Yuen WY (2014). Health-related quality of life in epidermolysis bullosa: validation of the Dutch QOLEB questionnaire and assessment in the Dutch population. Acta Derm. Venereol..

[16] Linertová R (2012). Delphi approach to select rare diseases for a European representative survey. The BURQOL-RD study. Health Policy.

[17] Drummond MF, O’Brien B, Stoddart GL, Torrance GW (1997). Methods for the economic evaluation of health care programmes.

[18] McDaid D (2001). Estimating the costs of informal care for people with Alzheimer’s disease: methodological and practical challenges. Int. J. Geriatr. Psychiatry.

[19] van den Berg B, Brouwer W, Koopmanschap M (2004). Economic valuation of informal care: an overview of methods and applications. Eur. J. Health Econ..

[20] Hodgson T, Meiners MR (1982). Cost-of-illness methodology: a guide to assessment practices and procedures. Milbank Mem. Fund. Q..

[21] Brooks R (1996). EuroQol: the current state of play. Health Policy.

[22] Collin C (1988). The Barthel ADL Index: a reliability study. Int. Disabil. Stud..

[23] Hébert R, Bravo G, Préville M (2000). Reliability, validity and reference values of the Zarit burden interview for assessing informal caregivers of community-dwelling older persons with dementia. Can. J. Aging/La Revue canadienne du vieillissement.

[24] Szende A, Janssen B, Cabases J (2014). Self-reported population health: an international perspective based on EQ-5D.

[25] Shah S, Vanclay F, Cooper B (1989). Improving the sensitivity of the Barthel index for stroke rehabilitation. J. Clin. Epidemiol..

[26] Frew JW, Murrell DF (2010). Quality of life measurements in epidermolysis bullosa: tools for clinical research and patient care. Dermatol. Clin..

[27] Angelis A, Tordrup D, Kanavos P (2015). Socio-economic burden of rare diseases: a systematic review of cost of illness evidence. Health Policy.

[28] Wodinsky HB (1984). Cost analysis of the Kozak protocol as used in an Ontario hospital in the treatment of children with epidermoly-sis bullosa. Can. Med. Assoc. J..

[29] Ramsay C (1984). Cost analysis of the Kozak protocol in the treatmentof epidermolysis bullosa. Can. Med. Assoc. J..

[30] Hubbard LD, Mayre-Chilton K (2015). Quality of life among adults with epidermolysis bullosa living with a gastrostomy tube since childhood. Qual. Health Res..

[31] Haynes L, Mellerio JE, Martinez AE (2012). Gastrostomy tube feeding in children with epidermolysis bullosa: consideration of key issues. Pediatr. Dermatol..

[32] Grocott P, Blackwell R, Weir H, Pillay E (2013). Living in dressings and bandages: findings from workshops with people with epidermolysis bullosa. Int. Wound J..

[33] Both H (2007). Critical review of generic and dermatology-specific health-related quality of life instruments. J. Invest. Dermatol..

[34] Fine JD (2005). Impact of inherited epidermolysis bullosa on parental interpersonal relationships, marital status and family size. Br. J. Dermatol..

[35] Ghatnekar, O., et al.: A literature review of instruments for measuring health-related quality of life in rare diseases, in Internal report of BURQOL-RD. The Swedish Institute for Health Economics (2011)

[36] Tordrup D, Mossman J, Kanavos P (2014). Responsiveness of the EQ-5D to clinical change: is the patient experience adequately represented?. Int. J. Technol. Assess. Health Care.

