# Supplementary material for: Social/economic costs and health-related quality of life in patients with epidermolysis bullosa in Europe
Source: Eur J Health Econ. 2016 Apr 23;17(Suppl 1):31–42. doi: 10.1007/s10198-016-0783-4 (PMC4869727; doi:10.1007/s10198-016-0783-4)
Supplement: Supplementary file 1 — Supplementary material 1 (DOCX 16 kb) [file 10198_2016_783_MOESM1_ESM.docx]

**Appendix I: BURQOL-RD Research Network**

- Canary Islands Foundation for Research and Health (FUNCIS) (Spain): Pedro Serrano-Aguilar, Renata Linertová
- Universidad Castilla-La Mancha (Spain): Julio López-Bastida, Juan Oliva-Moreno
- Research Institute for Rare Diseases, Instituto de Salud Carlos III (Spain): Manuel Posada de la Paz, Manuel Hens Pérez, Ignacio Abaitua
- National Center for Rare Diseases, Istituto Superiore di Sanità (Italy): Domenica Taruscio, Yllka Kodra
- Mario Negri Institute for Pharmacological Research (Italy): Arrigo Schieppati
- Bulgarian Association for Promotion of Education and Science (Bulgaria): Rumen Stefanov, Georgi Iskrov
- Centre for Public Affairs Studies Foundation (Hungary): László Gulácsi, Márta Péntek, Valentin Brodszky, Petra Baji
- Federación Española de Enfermedades Raras (Spain): Rosa Sánchez de Vega García, Claudia Delgado
- London School of Economics and Political Science (UK): Panos Kanavos, Aris Angelis, Elena Nicod
- Leibniz University Hannover (Germany): Johann-Matthias Graf von der Schulenburg, Alexander Kuhlmann
- The Swedish Institute for Health Economics (Sweden): Ulf Persson, Ola Ghatnekar
- University Paris Est (France): Karine Chevreul, Karen Brigham
- Universita Commerciale “Luigi Bocconi” (Italy): Giovanni Fattore, Marianna Cavazza
