# Supplementary material for: Social/economic costs and health-related quality of life in patients with epidermolysis bullosa in Europe
Source: Eur J Health Econ. 2016 Apr 23;17(Suppl 1):31–42. doi: 10.1007/s10198-016-0783-4 (PMC4869727; doi:10.1007/s10198-016-0783-4)
Supplement: Supplementary file 2 — Supplementary material 2 (DOC 41 kb) [file 10198_2016_783_MOESM2_ESM.doc]

**Annex II: Unit costs sources**

Bulgaria

- National Health Insurance Fund (all direct healthcare costs) - [http://www.nhif.bg](http://www.nhif.bg/)
- National Statistical Institute - [http://www.nsi.bg](http://www.nsi.bg/)

France

- Base des médicaments et informations tarifaires. Available from: <http://www.codage.ext.cnamts.fr/codif/bdm_it/index_presentation.php?p_site=AMELI>
- Liste des produits et prestations. Available from: <http://www.codage.ext.cnamts.fr/codif/tips/index_presentation.php?p_site=AMELI>
- Classification commune des actes médicaux. Available from: <http://www.ameli.fr/accueil-de-la-ccam/index.php>
- Table nationale de codage de biologie. Available from: <http://www.codage.ext.cnamts.fr/codif/nabm/index_presentation.php?p_site=AMELI>
- Echelle nationale coûts par GHM. Available from: <http://www.atih.sante.fr/?id=000370000AFF>
- Institut national de la statistique et des études économiques. Available from: <http://www.insee.fr/fr/>

Germany

- Outpatient physician visits/services: National Association of Statutory Health Insurance Physicians: Uniform Value Scale 2012.[http://www.kbv.de](http://www.kbv.de/)
- Inpatient services/hospitalization: InEK GmbH – Institute for the Hospital Remuneration System: Diagnosis Related Group-Catalogue 2012. Düsseldorf: Dt. Krankenhaus-Verl.-Ges; 2011
- Drugs: Lauertaxe: Drug prices. <https://www.lauer-fischer.de/LF/Seiten/Verwaltung/Kundencenter.aspx>.
- Productivity loss: Federal Statistical Office of Germany (Statistisches Bundesamt): VGR des Bundes - Arbeitnehmerentgelt, Löhne und Gehälter (2012). <https://www-genesis.destatis.de/genesis/online/data;jsessionid=9CC92C2529D2AEBA4D9B9585B3A07CE6.tomcat_GO_2_1?operation=abruftabelleBearbeiten&levelindex=2&levelid=1436786131937&auswahloperation=abruftabelleAuspraegungAuswaehlen&auswahlverzeichnis=ordnungsstruktur&auswahlziel=werteabruf&selectionname=81000-0007&auswahltext=&werteabruf=Werteabruf>
- Professional care: Volume XI of the Social Insurance Code: §§ 36-45. Stiftung Warentest: Finance test 2006 (4): 68-69.
- Materials: Medical and health care suppliers.

Hungary

- National Health Insurance Fund Administration in Hungary - <http://www.oep.hu/>
- Hungarian Central Statistics Office

Italy

- Fees of Regional Health Service of Regione Lombardia
- Pricing market analysis in private healthcare sector
- Italian Medicines Agency, National Drug Code
- National Collective Work Contract – Social Cooperatives, Agreed Text 2006 – 2009
- Pricing market analysis at Italian Local Healthcare Units and City Councils
- National Social Insurance Agency and Eurostat

Spain

- Drugs: Vademecum Internacional. Medicom S.A. 44ª ed. Madrid, 2004.
- Visits/exams: Oblikue Consulting. Base de Datos de Costes Sanitarios eSALUD Barcelona. Available at: <http://www.oblikue.com/bddcostes>.
- Productivity losses: Instituto de Mayores y Servicios Sociales (IMSERSO). Las personas mayores en España. Datos estadísticos estatales y por Comunidades Autónomas. Informe 2008. Ministerio de Sanidad y Política Social, Madrid, 2008. Available from: <http://www.jubiladosugt.org/documentos/estudios_sociales/informe_personas_mayores_08_tomo_01.pdf>.

Sweden

- Drugs: [www.apoteket.se](http://www.apoteket.se/)
- Other costs: Statistics Sweden. Available from: http://www.scb.se/Pages/SalariesSearch____259066.asp

UK

- Payment by Results in the NHS: tariff for 2012 to 2013. Available from: <https://www.gov.uk/government/publications/confirmation-of-payment-by-results-pbr-arrangements-for-2012-13>.
- NHS reference costs 2012 to 2013. Available from: <https://www.gov.uk/government/publications/nhs-reference-costs-2012-to-2013>.
- Curtis, L. Unit Costs of Health and Social Care 2012. Available from: <http://www.pssru.ac.uk/project-pages/unit-costs/2012/>.
- NHS Drug Tariff. Available from: <http://www.ppa.org.uk/ppa/edt_intro.htm>.
- British National Formulary. Available from: <http://www.bnf.org/bnf/index.htm>.
